# *APETALA2* antagonizes the transcriptional activity of *AGAMOUS* in regulating floral stem cells in *Arabidopsis thaliana*

**DOI:** 10.1111/nph.14151

**Published:** 2016-09-08

**Authors:** Zhigang Huang, Ting Shi, Binglian Zheng, Rae Eden Yumul, Xigang Liu, Chenjiang You, Zhihong Gao, Langtao Xiao, Xuemei Chen

**Affiliations:** ^1^ Hunan Provincial Key Laboratory of Phytohormones and Growth Development Hunan Provincial Key Laboratory for Crop Germplasm Innovation and Utilization Hunan Agricultural University Changsha 410128 China; ^2^ Department of Botany and Plant Sciences Institute of Integrative Genome Biology University of California Riverside CA 92521 USA; ^3^ College of Horticulture Nanjing Agricultural University No. 1 Weigang Nanjing 210095 China; ^4^ State Key Laboratory of Genetic Engineering Collaborative Innovation Center for Genetics and Development Institute of Plant Biology School of Life Sciences Fudan University Shanghai 200438 China; ^5^ State Key Laboratory of Plant Cell and Chromosome Engineering Center for Agricultural Resources Research Institute of Genetics and Developmental Biology Chinese Academy of Sciences Shijiazhuang 050021 China; ^6^ Guangdong Provincial Key Laboratory for Plant Epigenetics College of Life Sciences and Oceanography Shenzhen University Shenzhen 518060 China; ^7^ Howard Hughes Medical Institute University of California Riverside CA 92521 USA

**Keywords:** AGAMOUS, APETALA2, floral determinacy, floral stem cells, KNUCKLES, WUSCHEL

## Abstract

- *APETALA2* (*AP2*) is best known for its function in the outer two floral whorls, where it specifies the identities of sepals and petals by restricting the expression of *AGAMOUS* (*AG*) to the inner two whorls in *Arabidopsis thaliana*. Here, we describe a role of *AP2* in promoting the maintenance of floral stem cell fate, not by repressing *AG* transcription, but by antagonizing *AG* activity in the center of the flower.
- We performed a genetic screen with *ag‐10* plants, which exhibit a weak floral determinacy defect, and isolated a mutant with a strong floral determinacy defect. This mutant was found to harbor another mutation in *AG* and was named *ag‐11*. We performed a genetic screen in the *ag‐11* background to isolate mutations that suppress the floral determinacy defect. Two suppressor mutants were found to harbor mutations in *AP2*.
- While *AG* is known to shut down the expression of the stem cell maintenance gene *WUSCHEL* (*WUS*) to terminate floral stem cell fate, *AP2* promotes the expression of *WUS*.
- *AP2* does not repress the transcription of *AG* in the inner two whorls, but instead counteracts *AG* activity.

## Introduction

The angiosperm flower is an innovation that supports sexual reproduction and consists of four types of floral organ: sepal, petal, stamen and carpel. A flower is formed from a group of undifferentiated cells known as the floral meristem. Like the shoot apical meristem (SAM), floral meristems harbor stem cells in the central zone and descendants of the stem cells that give rise to organ primordia in the peripheral zone. In both the SAM and floral meristems, a small number of cells that express the *WUSCHEL* (*WUS*) gene lie underneath the stem cells (Mayer *et al*., 1998; Schoof *et al*., 2000). The WUS protein moves out of the cells that express the gene and forms a gradient towards the stem cells, where it activates the expression of the stem cell gene *CLAVATA3* to maintain stem cell fate (Yadav *et al*., 2011; Daum *et al*., 2014).

Unlike the SAM, which maintains stem cells throughout the life of a plant, floral meristems are determinate insofar as the stem cells are only active for a defined period during which the floral organ primordia are formed. When the final floral organ primordia, carpel primordia, are formed, the floral stem cells cease to be maintained, as evidenced by the absence of subsequent organ primordia and the cessation of *WUS* expression. The timing of floral stem cell fate termination is tightly coupled with organ formation. While a wild‐type *Arabidopsis thaliana* flower consists of four sepals, four petals, six stamens and two carpels, *wus* null mutant flowers have four sepals, four petals and a single stamen as a result of the premature termination of floral stem cell fate (Laux *et al*., 1996). In contrast, failure to terminate the floral stem cells results in additional floral organs or even flowers internal to the fourth whorl. Many genes, such as *AGAMOUS* (*AG*), *CRABS CLAW* (*CRC*), *KNUCKLES* (*KNU*), *ULTRAPETALA1* (*ULT1*), *CURLY LEAF* (*CLF*), *AUXIN RESPONSE FACTOR3* (*ARF3*), *POWERDRESS* (*PWR*) and *MICRORNA172d* (*MIR172d*), promote the termination of floral stem cell fate (Bowman *et al*., 1989; Bowman & Smyth, 1999; Carles *et al*., 2004; Zhao *et al*., 2007; Prunet *et al*., 2008; Sun *et al*., 2009; Liu *et al*., 2011, 2014a,b; Yumul *et al*., 2013). The central role of the MADS domain protein AG in conferring floral determinacy is well established, as loss‐of‐function *ag* mutants continually produce floral organs, resulting in a flowers‐within‐flower phenotype (Bowman *et al*., 1989). Mutations in the other genes result in weaker floral determinacy defects or only result in floral determinacy defects in combination with other mutations. *AG* promotes floral determinacy by shutting off *WUS* expression at stage 6 of flower development (Lenhard *et al*., 2001; Lohmann *et al*., 2001), both directly at the *WUS* locus and indirectly through its target gene *KNU* (Sun *et al*., 2009; Liu *et al*., 2011).

In addition to its critical role in floral determinacy, *AG* is a master regulator in floral organ identity specification. Together with other MADS domain proteins, AG specifies stamen identify in the third whorl and carpel identity in the fourth whorl (Bowman *et al*., 1991). In *ag* null mutants, stamens and carpels are replaced by petals and sepals, respectively (Bowman *et al*., 1989). *AG* expression is restricted to the inner two floral whorls by *APETALA2* (*AP2*) (Drews *et al*., 1991), which encodes an AP2 domain transcription factor (Jofuku *et al*., 1994). In *ap2* loss‐of‐function mutants, the ectopic expression of *AG* in the outer two whorls results in the transformation of sepals and petals into reproductive organs (Bowman *et al*., 1991; Drews *et al*., 1991). *AP2* also plays a role in stem cell maintenance in the SAM independent of its function in the repression of *AG* expression (Wurschum *et al*., 2006). *AP2*, a target of miR172 (Aukerman & Sakai, 2003; Chen, 2004), has also been implicated in the control of floral stem cells. The expression of an miR172‐resistant version of *AP2* driven by its own promoter results in prolonged *WUS* expression and an indeterminate flower (Zhao *et al*., 2007). However, a role of *AP2* in promoting floral stem cell fate is not obvious in *ap2* loss‐of‐function mutants, which have fewer floral organs in the outer three whorls but the normal number of carpels (Bowman *et al*., 1989, 1991). The reduced organ number in the outer three whorls is probably linked to *AP2*'s role in repressing *AG* expression, as *ap2 ag* double mutants have normal numbers of organs in the outer three whorls (Bowman *et al*., 1989, 1991). Thus, there is no clear loss‐of‐function genetic evidence supporting a role of *AP2* in maintaining floral stem cells in the center of the flower.

Through its function as a master regulator of both organ identity and floral determinacy, *AG* coordinates various cell fate decisions in flower development. However, this raises a logistical problem: while *AG* is expressed in the inner two whorls from stage 3 and onward to specify the identities of the reproductive organs, its repression of *WUS* must not commence until stage 6 when the carpel primordia have formed. A timing mechanism involving the delayed activation of *KNU* by *AG* at stage 6 has been described (Sun *et al*., 2009, 2014), but it remains unknown whether other factors contribute to the timing of *KNU* expression. One possibility is that *AG*'s activation of *KNU* and/or repression of *WUS* is kept in check by a negative regulator. Here we show that *AP2* antagonizes *AG* in the regulation of *KNU*,* WUS* and many early floral patterning genes. In this study, we isolated an intermediate‐strength *ag* allele, *ag‐11*, which compromises the floral determinacy but not the organ identity functions of *AG*. We then performed a genetic screen in the *ag‐11* background and isolated mutations that suppressed the floral determinacy defect. Two were found to harbor mutations in *AP2*. We showed that *AP2* did not affect the levels of *AG* transcripts in the inner two floral whorls, but instead antagonized *AG* in terms of the control of *WUS* repression. Moreover, *AP2* antagonized *AG* activity in the inner two whorls even when *AG* was expressed from the cauliflower mosaic virus 35S promoter, which is not controlled by *AP2*. Therefore, we have uncovered a previously unappreciated function of *AP2* as a braking mechanism in the termination of floral stem cell maintenance. Our findings on floral stem cell regulation in the center of the flower also hint at a more complex relationship between *AP2* and *AG* beyond what the canonical model of flower development (i.e. ABC) (Bowman *et al*., 1991) and updated versions (Wollmann *et al*., 2010) suggest.

## Materials and Methods

### Plant material

All mutants and transgenic lines used in this study are in the *Arabidopsis thaliana* Landsberg *erecta* (L*er*) background. All plants were grown at 23°C under long‐day conditions (16 h : 8 h, light : dark). *ap2‐2* (Bowman *et al*., 1991), *ag‐10* (Ji *et al*., 2011), *ag‐10* ^*col*^ (Liu *et al*., 2011), *35S::AG* (Mizukami & Ma, 1992) and *35S::AP2m3* (Chen, 2004) were previously described.

### Ethyl methane sulfonate (EMS) mutagenesis and map‐based cloning

Approximately 10 000 *ag‐10* and *ag‐11* seeds were washed with 0.1% Tween 20 for 15 min, incubated with ethyl methane sulfonate (EMS; 0.2% w/v) for 12 h at room temperature and then washed three times with 10 ml of water (1 h for each wash on a rotator). The treated seeds were grown into M1 plants, and M2 seeds were harvested from the M1 plants for genetic screening. *ag‐10* enhancers were isolated based on the presence of bulged siliques throughout the plant, and *ag‐11* suppressors were isolated based on the suppression of the bulged‐silique phenotype. The mutants were backcrossed at least two times before further study. For map‐based cloning, *ag‐11* was crossed with *ag‐10* ^*Col*^ to create the mapping population. Simple sequence length polymorphism (SSLP) and cleaved‐amplified polymorphic sequence (CAPS) markers were used to map the mutations. Once *ag‐11* was mapped to an interval containing the *AG* locus, *AG* was selected as a candidate gene for sequencing. For *ag‐11* suppressors *B35* (*ag‐11 ap2‐35*) and *B43* (*ag‐11 ap2‐43*), *AP2* was selected as a candidate gene for sequencing based on the similarity of their phenotypes to *ap2* loss‐of‐function mutants.

### DNA isolation and genotyping

Two methods, cetyl trimethyl ammonium bromide DNA extraction and ‘quick & dirty’ extraction, were used for DNA isolation as described previously (Dinh *et al*., 2014). The following primer pairs and enzymes were used for genotyping: *ag‐10* (JAGp75 and JAGp76; *Bst*XI), *ag‐11* (ag‐11F and ag‐11R; *Hin*fI) and *ag‐1* (ag‐1F and ag‐1R; *Afl*II). In each case, the mutations abolish the targeted restriction site. The sequences of the PCR primers are listed in Supporting Information Table S1.

### Scanning electron microscopy

Scanning electron microscopy (SEM) imaging was performed with a Hitachi TM‐1000 tabletop scanning electron microscope (Hitachi, Tokyo, Japan) according to the manufacturer's instructions. Samples imaged by the TM‐1000 require no special preparation.

### *In situ* hybridization

*In situ* hybridization was performed as previously described (Chen *et al*., 2002). To generate the antisense *AG* probe, the plasmid pCIT565 (Yanofsky *et al*., 1990) was linearized with *Hin*dIII and used as a template for *in vitro* transcription with T7 RNA polymerase. To generate the *WUS* probe, a plasmid containing the *WUS* cDNA was used as the PCR template to generate products containing either the T7 or SP6 promoter sequence. *In vitro* transcription was performed with either T7 or SP6 RNA polymerase using the purified PCR product as the template to generate the antisense or sense probe, respectively, as previously described (Liu *et al*., 2011). The PCR primers are listed in Table S1.

### RNA isolation and real‐time RT‐PCR

Total RNA was extracted from inflorescences containing stage 7 and younger flowers using TRI reagent (MRC, Cincinnati, OH, USA), and DNA was removed using DNase I (Roche). RevertAid Reverse Transcriptase (Thermo Fisher Scientific, Waltham, MA, USA) was used to synthesize cDNA. Quantitative PCR was performed in triplicate using the Bio‐Rad CFX‐96 Real‐time PCR system and iQ SYBR Green Supermix (Bio‐Rad). All procedures were performed according to the manufacturers' instructions. The primers used for real‐time reverse transcription−polymerase chain reaction (RT‐PCR) are listed in Table S1.

### RNA‐seq analysis

Total RNA was extracted from inflorescence tissue containing stage 7 and younger flowers using TRI reagent (MRC). Five micrograms of total RNA was used to isolate mRNA using the NEBNext^®^ Poly(A) mRNA Magnetic Isolation Module (NEB, Ipswich, MA, USA). RNA‐seq libraries were constructed using the NEBNext^®^ mRNA Library Prep Reagent Set for Illumina (NEB) following the manufacturer's protocols. Twelve libraries (three replicates for each sample) were pooled and sequenced on an Illumina HiSeq 2500 (Illumina, San Diego, CA, USA) platform at the UCR Genomics Core Facility. Reads that passed Illumina's quality control filters were further processed. Unique reads were mapped to the L*er* genome (Gan *et al*., 2011) using tophat v.2.0.13 (Kim *et al*., 2013), with no mismatches permitted. Reads in gene regions were counted using an in‐house Perl script. The expression fold‐change of each gene was calculated using the R package DESeq2 (Love *et al*., 2014) between L*er* and *ag‐11*,* ag‐11* and *ag‐11 ap2‐35*, and *ag‐11* and *ag‐11 ap2‐43*, with the threshold for differentially expressed (DE) genes set to a fold‐change of 1.5 and a *P*‐value < 0.01. Venn diagrams were generated using Venny v.2.1 (http://bioinfogp.cnb.csic.es/tools/venny/index.html), and the gene ontology (GO) enrichment analysis of DE genes was performed on the agriGO website (Du *et al*., 2010). AG and AP2 binding sites were extracted from published chromatin immunoprecipitation (ChIP)‐seq data (Yant *et al*., 2010; Ó'Maoiléidigh *et al*., 2013) and mapped to the arabidopsis information resource v.10 (TAIR10; https://www.arabidopsis.org) genome. Genes with binding sites within the gene body and 1000‐bp flanking sequences were designated as genes bound by AG or AP2 *in vivo*.

To assess the statistical significance of the overlap of DE genes between pairs of samples, a chi‐squared test was performed with 10 000 iterations of overlap analysis between randomly generated gene sets containing the same numbers of genes as the DE genes.

The RNA‐seq data were deposited in GEO under the accession number GSE81205. The link for reviewers: http://www.ncbi.nlm.nih.gov/geo/query/acc.cgi?token=uxyjsicqdxwbjkp&acc=GSE81205.

### Western blotting

Western blotting was performed as previously described (Liu *et al*., 2011). One hundred milligrams of inflorescence tissue from each sample was ground in liquid nitrogen and homogenized in 2×  sodium dodecyl sulfate (SDS) sample buffer (0.5 M Tris‐HCl, pH 6.8, 4.4% (w/v) SDS, 20% (v/v) glycerol, 2% (v/v) 2‐mercaptoethanol, and bromophenol blue). The samples were boiled for 6 min, cooled on ice for 10 min and centrifuged at 16 000 ***g*** for 5 min at 4°C to precipitate insoluble material. Proteins in the supernatant were resolved on a 12% sodium dodecyl sulfate−polyacrylamide gel electrophoresis (SDS‐PAGE) gel, transferred to a nitrocellulose membrane and probed with anti‐AG (Liu *et al*., 2011), anti‐AP2 (Mlotshwa *et al*., 2006) and anti‐HSC70 (Santa Cruz Biotechnology, Santa Cruz, CA, USA) antibodies. Signal development was performed with the ECL+Plus Western Blotting system (GE Healthcare, Pasadena, CA, USA) and by exposure of the membrane to X‐ray film (Denville, Holliston, MA, USA).

## Results

### Isolation of *ag‐11*, an *ag* allele that uncouples the organ identify and floral determinacy functions of *AG*

**Figure 1:**
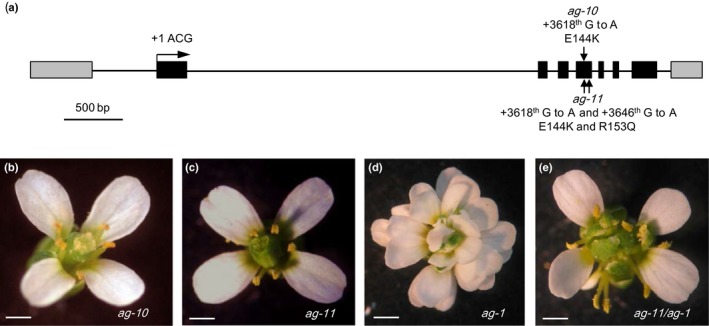
Diagram of the *Arabidopsis thaliana AGAMOUS* (*AG*) gene and phenotypes of *ag* mutants. (a) Gene diagram of *AG* and the locations of the mutations in *ag‐10* and *ag‐11*. ACG is the start codon. The gray and black rectangles represent the 5′ and 3′ untranslated regions and coding regions, respectively. The black lines represent intron regions. (b) An *ag‐10* flower with a slightly enlarged gynoecium. (c) An *ag‐11* flower with a much more enlarged gynoecium compared with *ag‐10*. (d) An *ag‐1* flower exhibiting the flower‐within‐flower phenotype. (e) An *ag‐11*/*ag‐1* flower with additional organs within the primary carpels, which are unfused and sepalloid. (b–e) Bars, 1 mm.

We previously reported the isolation and characterization of the *ag‐10* mutant, which harbors a point mutation resulting in an E‐to‐K amino acid substitution in the K domain of AG (Fig. 1a) (Ji *et al*., 2011). This mutant is the weakest *ag* allele reported thus far. As in wild type, *ag‐10* flowers have a full complement of floral organs (Fig. 1b). Most *ag‐10* flowers are normal in terms of floral determinacy; < 10% of *ag‐10* flowers have bulged gynoecia with additional floral organs inside, a manifestation of compromised floral determinacy (Ji *et al*., 2011). We previously mutagenized *ag‐10* seeds with EMS and conducted a genetic screen for mutants with enhanced floral determinacy defects. Mutants in which nearly all flowers had bulged gynoecia were isolated, and some have been reported (Liu *et al*., 2011, 2014a,b; Yumul *et al*., 2013).

**Figure 2:**
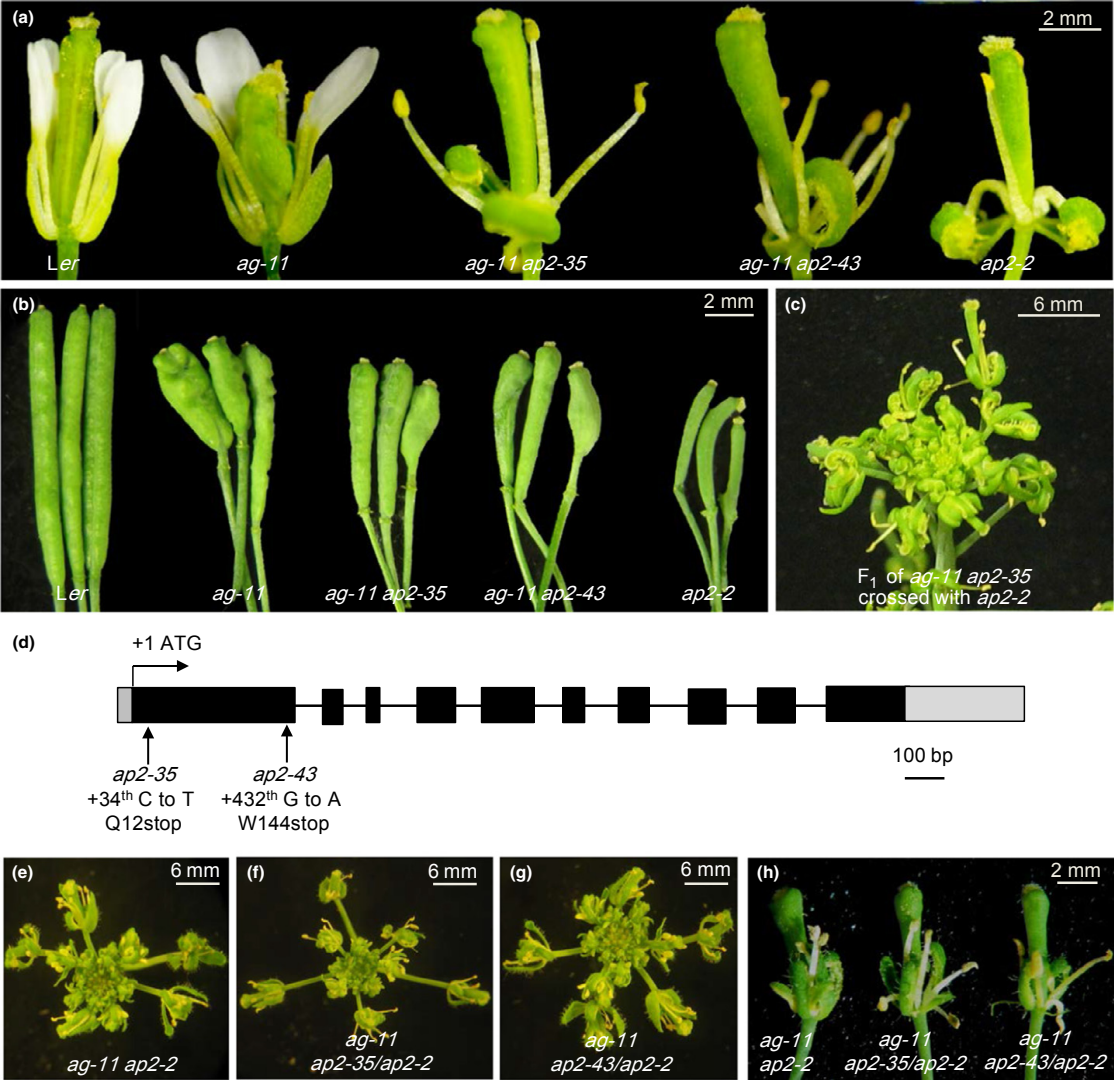
Diagram of the *Arabidopsis thaliana APETALA2* (*AP2*) gene and phenotypes of *ag‐11* and *ap2* single and double mutants. Note that B35 and B43 are *ag‐11 ap2‐35* and *ag‐11 ap2‐43*, respectively. (a) Flowers from plants of the indicated genotypes. The flowers from *ag‐11 ap2‐35* and *ag‐11 ap2‐43* had longer and thinner gynoecia compared with *ag‐11* flowers. (b) Siliques from plants of the indicated genotypes. (c) An inflorescence from F_1_ plants of the cross between *ag‐11 ap2‐35* and *ap2‐2*. The flowers were similar in morphology to those of *ap2‐2*. (d) Gene diagram of *AP2* showing the locations of the *ap2* mutations. ATG is the start codon. The gray and black rectangles represent the 5′ and 3′ untranslated regions and coding regions, respectively. The black lines represent introns. (e) An *ag‐11 ap2‐2* inflorescence. (f, g) Inflorescences from F_1_ plants of the cross *ag‐11 ap2‐2 *×* ag‐11 ap2‐35* and *ag‐11 ap2‐2 *×* ag‐11 ap2‐43*, respectively. The flowers were similar in morphology to those of *ag‐11 ap2‐2*. (h) Flowers of the indicated genotypes showing that the siliques were long and thin.

Here, we report another mutant from this genetic screen. This mutant had bulged gynoecia in nearly all of its flowers (Figs 1c, 2a,b) and elongated gynophores (Fig. 2b), another feature found in many mutants compromised in floral determinacy. Unlike the previously reported mutants from the *ag‐10* genetic screen, such as *clf*,* arf3*,* topoisomerase1α* (*top1α*), *pwr* and *mir172d*, which exhibited other developmental defects (in flowering time, leaf development, etc.) (Liu *et al*., 2011, 2014a,b; Yumul *et al*., 2013), this mutant did not have any defects other than floral determinacy.

To map this mutation, we crossed the mutant to *ag‐10* ^*Col*^, in which the *ag‐10* mutation was introgressed into the Columbia (Col) background. Using markers polymorphic between L*er* and Col, we mapped the mutation to the short arm of chromosome 4 to an interval containing the *AG* locus. Sequencing of the *AG* gene itself uncovered a G‐to‐A mutation that causes an R‐to‐Q amino acid substitution in the K domain (Fig. 1a). This *ag* allele harboring both the original *ag‐10* mutation and this new mutation was designated *ag‐11*. To determine whether the phenotype is attributable to the mutations in *AG*, we performed a genetic complementation test. The *ag‐11* mutant was crossed with an *ag‐1* heterozygous plant. In a total of nine F1 plants, five exhibited an intermediate phenotype that was stronger than *ag‐11* and weaker than *ag‐1* (Fig. 1e), and the other four resembled wild type (Fig. S1). Molecular genotyping showed that the five plants were *ag‐1*/*ag‐11*, and the four plants were *ag‐11*/+. Progeny of the four plants all segregated *ag‐11*‐like phenotypes. Together, these observations indicated that this mutant is an *ag* allele.

The *ag‐11* allele differs from *ag* null alleles (*ag‐1* or *ag‐3*) (Bowman *et al*., 1989, 1991) in that it uncouples the organ identity and floral determinacy functions of *AG*. While *ag* null mutants feature indeterminate flowers that also lack reproductive organs, the *ag‐11* mutant had indeterminate flowers with the correct types of organ in all four whorls (Fig. 1c). The organ numbers in the outer three whorls in *ag‐11* were normal, and carpel number was slightly increased (Table S2). Consistent with the largely unaffected floral organ identities in *ag‐11* flowers, it is not surprising that the *ag‐11* mutant is fertile. An allelic series of *ag* mutants now exists (listed in order of increasing phenotypic severity): *ag‐10* (Ji *et al*., 2011), *ag‐11*,* ag‐4* (Sieburth *et al*., 1995) and *ag‐1* (or *ag‐3*) (Bowman *et al*., 1989, 1991). It is worth noting the differences between *ag‐11* and *ag‐4*: the former retains stamen and carpel identities, while the latter lacks carpel identity and is thus female sterile. The *ag‐11*/*ag‐1* plants (Fig. 1e) were strikingly similar to *ag‐4*, suggesting that floral phenotypes are sensitive to the dosage of *AG* functions.

### Mutations in *AP2* partially suppress the floral determinacy defect of *ag‐11*

The fertility of *ag‐11* allowed a genetic screen in this background with relative ease. Theoretically, loss‐of‐function mutations that suppress the floral determinacy defect of *ag‐11* should be in genes that promote floral stem cell fate. A major consideration for performing the *ag‐11* genetic screen was the fact that *WUS* is the only gene presently known to promote floral stem cell fate. Thus, we mutagenized *ag‐11* with EMS and screened for mutations that suppressed the floral determinacy defect.

Two mutants (*B35* and *B43*) with longer and thinner gynoecia were isolated (Fig. 2a,b). Backcrosses to *ag‐11* showed that each mutant harbored a recessive mutation (Fig. S2a). F1 plants from crosses of the two mutants to each other had longer and thinner gynoecia as compared with *ag‐11* (Fig. S2a,b), suggesting that these two mutants harbored mutations in the same gene. These mutants also had other floral defects, including reduced floral organ numbers in whorls 1 and 3, carpelloid sepals and a lack of petals (Fig. 2a; Table S2). These phenotypes are characteristic of *ap2* loss‐of‐function mutants (Fig. 2a) (Bowman *et al*., 1991). When *B35* and *B43* were crossed to *ap2‐2*, the F_1_ plants resembled *ap2‐2* plants in overall floral morphology (Fig. 2c), indicating that *B35* and *B43* harbor mutations in *AP2*. Sequencing of *AP2* in the two mutants revealed G‐to‐A mutations resulting in premature stop codons in exon 1 of *AP2* (Fig. 2d). As the two mutations introduce premature stop codons close to the N‐terminus of AP2, it is likely that they are loss‐of‐function mutations. To determine whether loss of function in *AP2* was responsible for suppressing the *ag‐11* floral determinacy defect, we introduced the loss‐of‐function *ap2* allele, *ap2‐2* (Bowman *et al*., 1991), into *ag‐11*. As with *B35* and *B43*, the *ag‐11 ap2‐2* double mutant exhibited an overall floral phenotype similar to that of *ap2* mutants (Fig. 2e). The siliques of *ag‐11 ap2‐2* were long and thin; thus, *ap2‐2* was also able to suppress the bulged silique phenotype of *ag‐11* (Fig. 2h). In addition, we conducted a genetic complementation test by crossing *B35* and *B43* to *ag‐11 ap2‐2*. The F_1_ progenies from each cross all resembled *ap2* mutants in overall floral morphology (Fig. 2f,g) and had longer and thinner gynoecia compared with *ag‐11* (Fig. 2h). We therefore concluded that *AP2* loss of function was responsible for the suppression of *ag‐11*. We refer to the two new *ap2* alleles as *ap2‐35* and *ap2‐43*, respectively.

**Figure 3:**
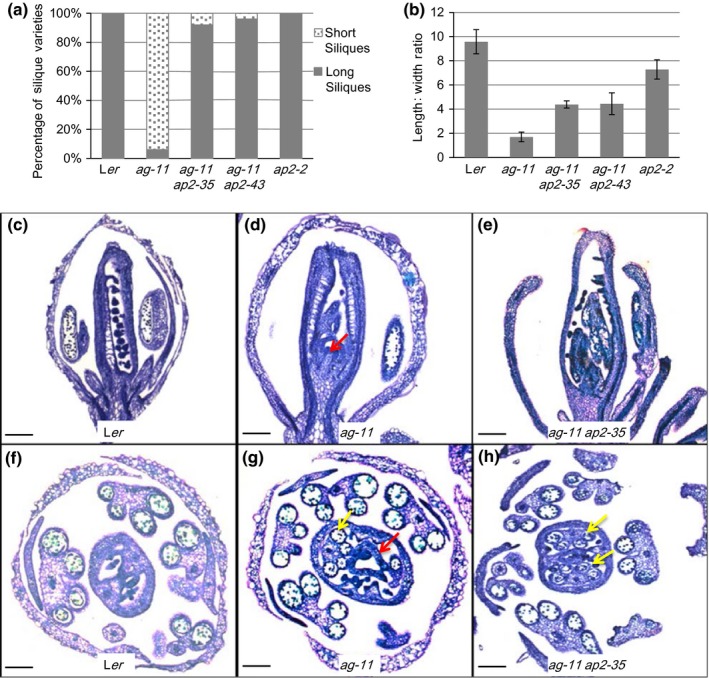
Characterization of floral determinacy phenotypes of various *Arabidopsis thaliana* mutants. (a) Silique length and (b) silique length : width ratio in *ag‐11* and the *ap2* single and double mutants. Short siliques, length ≤ 4 mm; long siliques, length > 4 mm. Fifty siliques were measured. Error bars in (b) represent ± SE. (c–e) Longitudinal sections of stage 11 or older flowers of the indicated genotypes. Wild‐type (L*er*) stage 11 flowers have gynoecia with ovules, and the floral meristem is not visible inside the gynoecium (c). A floral meristem was found internal to the gynoecium near the base in *ag‐11* (d; red arrow) but not in *ag‐11 ap2‐35* (e). (f–h) Cross‐sections of stage 11 or older flowers of the indicated genotypes. Anthers were present inside the fourth whorl gynoecium in *ag‐11* (g, yellow arrow) and *ag‐11 ap2‐35* (h, yellow arrows). A meristem‐generating organ primordium is also visible inside the gynoecium in *ag‐11* (g, red arrow). Bars, 50 μm.

While most *ag‐11* gynoecia were short and bulged, most *ag‐11 ap2‐35* and *ag‐11 ap2‐43* flowers had longer, thin and straight gynoecia (Fig. 3a). Quantification of the silique length:width ratio showed that the two *ap2* alleles partially suppressed the short‐and‐bulged silique phenotype of *ag‐11* (Fig. 3b). To further characterize the floral determinacy phenotype, we performed longitudinal and cross sections of stage 11 and older flowers. In the wild type, stage 11 flowers had gynoecia with ovules, and the floral meristem was not visible inside the gynoecium at this stage (Fig. 3c). In stage 11 *ag‐11* flowers, a floral meristem was present within the gynoecium near the base (Fig. 3d). In a cross section, it was obvious that additional floral organs were present inside the fourth whorl gynoecium (Fig. 3g); this was never observed in the wild type (Fig. 3f). In *ag‐11 ap2‐35*, 24 out of 26 flowers examined resembled *ap2‐2* flowers, while two out of 26 flowers examined had floral organs inside the gynoecium (Fig. 3e,h). These observations show that the two *ap2* alleles partially suppressed the *ag‐11* floral determinacy defect.

### *AP2* promotes *WUS* expression without affecting *AG* transcription in the center of floral meristems

**Figure 4:**
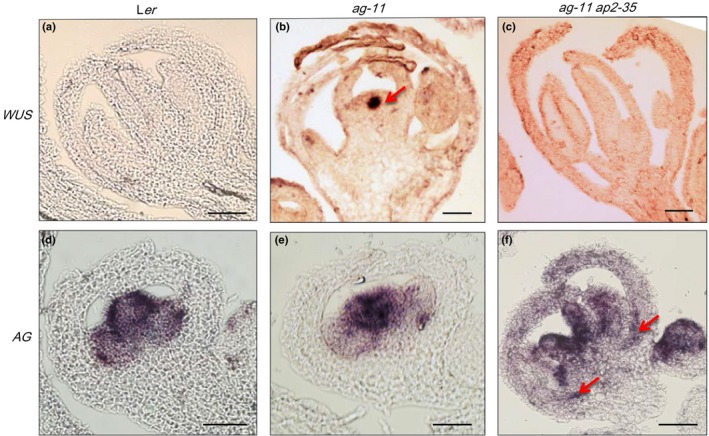
Expression of *Arabidopsis thaliana WUSCHEL* (*WUS*) and *AGAMOUS* (*AG*) as determined by *in situ* hybridization. (a–c) *WUS* expression in various genotypes. (a) A stage 9 wild‐type (L*er*) flower with no *WUS* expression. (b) A stage 9 *ag‐11* flower; *WUS* expression is indicated by the arrow. (c) A stage 10 *ag‐11 ap2‐35* flower with no *WUS* expression. (d–f) *AG* expression in stage 6–7 flowers of various genotypes. *AG* expression was observed in the two inner whorls in (d) L*er*, (e) *ag‐11* and (f) *ag‐11 ap2‐35*. *AG* expression was also detected in sepals, especially at the base, in *ag‐11 ap2‐35* (f, arrows). Bars, 50 μm.

We next sought to determine the molecular basis of the suppression of *ag‐11* phenotypes by *ap2* mutations. We first examined *WUS* expression by *in situ* hybridization, as *AG*‐mediated cessation of *WUS* expression by stage 6 of flower development is responsible for floral determinacy. Consistent with previous observations in wild type (Mayer *et al*., 1998), *WUS* expression was detected in stage 3 floral meristems in a small number of cells underneath the stem cells, but *WUS* expression was not detected in stage 6 or older flowers (Fig. 4a). In *ag‐11* plants, however, all examined stage 6 and older flowers had *WUS* expression (*n *=* *12) (Fig. 4b). Prolonged *WUS* expression is consistent with the continued presence of a floral meristem inside the gynoecium in *ag‐11* (Fig. 3d). In *ag‐11 ap2‐35*, 11 out of 15 stage 6 or older flowers examined resembled wild type in having no *WUS* expression (Fig. 4c). The remaining four resembled *ag‐11*. Loss of function in *AP2* therefore partially suppressed *ag‐11* in terms of the *WUS* repression defect; in other words, the prolonged *WUS* expression in *ag‐11* flowers required *AP2* function.

**Figure 5:**
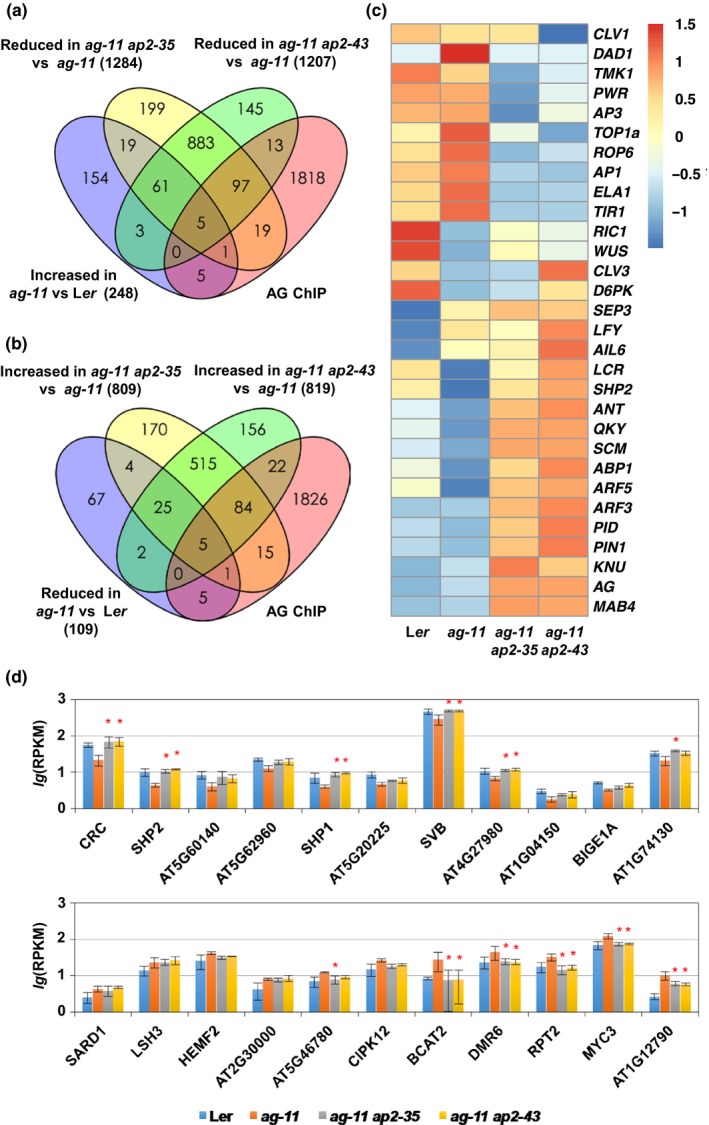
Antagonistic effects of *Arabidopsis thaliana AGAMOUS* (*AG*) and *APETALA2* (*AP2*) on target gene expression as determined by RNA‐seq. (a) Venn diagram showing the overlap between genes with increased expression in *ag‐11* vs L*er* and genes with reduced expression in *ag‐11 ap2‐35* vs *ag‐11* or *ag‐11 ap2‐43* vs *ag‐11*. (b) Venn diagram showing the overlap between genes with reduced expression in *ag‐11* vs L*er* and genes with increased expression in *ag‐11 ap2‐35* vs *ag‐11* or *ag‐11 ap2‐43*. In (a) and (b), genes with AG binding sites as determined by AG chromatin immunoprecipitation (ChIP)‐seq (Ó'Maoiléidigh *et al*., 2013) are also shown. The numbers in parentheses represent the numbers of genes with altered expression between the two indicated genotypes. (c) Expression levels of selected differentially expressed (DE) genes in *ag‐11* vs L*er* with known roles in early flower development. The differential expression of these genes in *ag‐11* was restored by the mutations in *AP2*. The heatmap was generated with *Z*‐score values derived from *Z* score = (*x* – mean)/SD (*x* being one of the four genotypes; mean and SD being calculated from the four genotypes). (d) Reads per kilobase per million mapped reads (RPKM) values for select *AG* target genes (genes bound by AG *in vivo*) in the indicated genotypes. The mean and SD from three biological replicates of RNA‐seq are shown. The asterisks indicate statistically significant changes relative to *ag‐11*. Fold change ≥ 1.5 and *P*‐value < 0.01. *CLV1*,* CLAVATA1*;* DAD1*,* DEFECTIVE ANTHER DEHISCENCE 1*;* TMK1*,* TRANSMEMBRANE KINASE 1*;* PWR*,* POWERDRESS*;* AP3*,* APETALA3*;* TOP1*α, *DNA TOPOISOMERASE I ALPHA*;* ROP6*,* Rho‐like GTPase 6*;* AP1*,* APETALA1*;* ELA1*,* EUI‐LIKE P450 A1*;* TIR1*,* TRANSPORT INHIBITOR RESPONSE 1*;* RIC1*,* ROP‐INTERACTIVE CRIB MOTIF‐CONTAINING PROTEIN 1*;* WUS*,* WUSCHEL*;* CLV3*,* CLAVATA3*;* D6PK*,* D6 PROTEIN KINASE*;* SEP3*,* SEPALLATA3*;* LFY3*,* LEAFY 3*;* AIL6*,* AINTEGUMENTA‐LIKE 6*;* LCR*,* LEAF CURLING RESPONSIVENESS*;* SHP2*,* SHATTERPROOF 2*;* ANT*,* AINTEGUMENTA*;* QKY*,* QUIRKY*;* SCM*,* SCRAMBLED*;* ABP1*,* AUXIN BINDING PROTEIN 1*;* ARF5*,* AUXIN RESPONSE FACTOR 5*;* ARF3*,* AUXIN RESPONSE FACTOR 3*;* PID*,* PINOID*;* PIN1*,* PIN‐FORMED 1*;* KNU*,* KNUCKLES*;* AG*,* AGAMOUS*;* MAB4*,* MACCHI‐BOU 4*;* CRC*,* CRABS CLAW*;* SHP1*,* SHATTERPROOF 1*;* SVB*,* SMALLER WITH VARIABLE BRANCHES*;* SARD1*,* SAR DEFICIENT 1*;* LSH3*,* LIGHT SENSITIVE HYPOCOTYLS 3*;* CIPK12*, CBL‐*INTERACTING PROTEIN KINASE 12*;* BCAT2*,* BRANCHED‐CHAIN AMINO ACID TRANSAMINASE 2*;* DMR6*,* DOWNY MILDEW RESISTANT 6*;* RPT2*,* ROOT PHOTOTROPISM 2*;* MYC3*,* MYC TRANSCRIPTION FACTOR 3*.

As *AG* is the key factor that represses *WUS* expression in stage 6 floral meristems, we next examined whether *AP2*'s role in promoting *WUS* expression was attributable to the ability of *AP2* to repress *AG* expression in the center of the flower. *AP2* is known to repress *AG* transcription in the outer two floral whorls (Drews *et al*., 1991), but it is thought not to repress *AG* transcription in the center of the floral meristem, as ectopic *AP2* activity in *ag‐1* was not sufficient to prevent the accumulation of *ag‐1* transcript in the inner whorls of *ag‐1* mutant flowers (Gustafson‐Brown *et al*., 1994). First, we assessed whether *AG* transcript levels were affected by the *ag‐11* mutation or the *ap2* mutations. RNA‐seq using inflorescences containing stage 7 and younger flowers from *ag‐11*,* ag‐11 ap2‐35* and *ag‐11 ap2‐43* showed increased *AG* transcripts in *ag‐11 ap2‐35* and *ag‐11 ap2‐43* compared with *ag‐11* (Fig. 5c). The increase in *AG* expression was probably attributable to the known repression of *AG* transcription in the outer two whorls by *AP2*. To determine whether *AP2* repressed *AG* transcription in the center of the floral meristem, where floral determinacy takes place, we examined *AG* expression by *in situ* hybridization in developing flowers. *AG* transcripts were present in the center of floral meristems but excluded from sepal primordia in wild type and *ag‐11* (Fig. 4d,e). In *ag‐11 ap2‐35*,* AG* RNA was detected not only in the center of the floral meristem but also in sepals (Fig. 4f). Although *in situ* hybridization is not quantitative, *AG* RNA signals were similar in *ag‐11* and *ag‐11 ap2‐35* in the center of the floral meristem (Fig. 4f). Therefore, the effect of the *ap2* mutations on *WUS* expression could not be attributed to obvious changes in *AG* transcription in the center of the floral meristem.

### *AP2* and *AG* have opposite transcriptional outputs at the genomic scale

*AP2* clearly exerts the opposite effect on *WUS* expression and floral determinacy compared with *AG*, and yet it does not appear to affect *AG* transcription in the center of the floral meristem. This suggests that *AP2* antagonizes *AG* activity, perhaps by preventing *AG* from acting on its target genes or acting independently on the same target genes to exert the opposite effect. To determine whether *AP2* and *AG* have antagonistic effects on transcription, we examined the gene expression profiles of L*er*,* ag‐11*,* ag‐11 ap2‐35* and *ag‐11 ap2‐43* using RNA‐seq. Inflorescences containing stage 7 and younger flowers were used, and three biological replicates were performed. For each genotype, the three biological replicates were highly correlated (Fig. S3a), indicating that the RNA‐seq data were reproducible. Using DESeq2, we identified DE genes for each pairwise comparison using fold‐change > 1.5 and *P*‐value < 0.01 as the threshold (Tables S3, S4). Compared with L*er*, 248 up‐regulated and 109 down‐regulated genes were found in *ag‐11* (Table S3). As expected, genes involved in plant development, especially flower development, were among the most significantly enriched in the down‐regulated genes (Fig. S3c). Intriguingly, genes involved in immune responses and cell death were significantly enriched in the up‐regulated genes (Fig. S3b). Eleven of the 248 up‐regulated genes and 11 of the 109 down‐regulated genes had AG‐binding sites, as determined by AG ChIP‐seq (Ó'Maoiléidigh *et al*., 2013), and may thus be direct targets of *AG* (Fig. 5a,b; Table S3). The other DE genes may be indirectly regulated by *AG*.

Remarkably and rather unexpectedly, the differential expression of a large portion of the 248 and 109 genes in *ag‐11* was rescued by the two *ap2* mutations. Among the 248 genes showing increased expression in *ag‐11* vs L*er*, 86 and 69 overlapped with genes with reduced expression in *ag‐11 ap2‐35* vs *ag‐11* and *ag‐11 ap2‐43* vs *ag‐11*, respectively (Fig. 5a). The overlap was statistically significant (see Table S5 for *P*‐values). Thus, *AP2* is required for the increased expression of these genes in the *ag‐11* mutant. Among the 109 genes showing reduced expression in *ag‐11*, 35 and 32 overlapped with the genes up‐regulated in *ag‐11 ap2‐35* vs *ag‐11* and *ag‐11 ap2‐43* vs *ag‐11*, respectively (Fig. 5b; see Table S5 for *P*‐values), indicating that *AP2* is required for the reduced expression of these genes in *ag‐11*. Among genes co‐regulated by *AG* and *AP2* were genes with roles in early flower development described in a previous review article (Vaddepalli *et al*., 2015), and the altered expression of these genes in *ag‐11* was restored by the mutations in *AP2* (Fig. 5c). Of particular note was that genes with previously established roles in floral stem cell regulation, such as *WUS*,* KNU*,* PWR*, and *ARF3*, were among the genes co‐regulated by *AG* and *AP2* in opposite directions (Fig. 5c). These findings show that *AP2* antagonizes the transcriptional activity of *AG* in the control of floral determinacy and perhaps in other aspects of flower development. It is worth noting that a much larger group of genes was affected by the *ap2* mutations (*ag‐11 ap2* vs *ag‐11*) than by *ag‐11* (*ag‐11* vs L*er*). Ectopic *AG* expression in the outer two whorls in *ap2* mutants could have contributed to a portion of the differentially expressed genes in *ag‐11 ap2* vs *ag‐11*.

We next examined whether *AP2* has an effect on direct targets of *AG*. Among the 11 up‐regulated and 11 down‐regulated direct *AG* targets showing differential expression in *ag‐11* vs L*er*, five of each category showed differential expression in the two *ap2* mutants in the opposite direction (Fig. 5a,b). That is, the altered expression of these *AG* target genes in *ag‐11* was restored by the mutations in *AP2* (some of the genes are shown in Fig. 5d). These findings indicate that *AP2* exerts the opposite effect on *AG* direct target genes. We asked whether these *AG* target genes are also potential *AP2* direct targets. Using published AP2 ChIP‐seq data (Yant *et al*., 2010), we found that two (*SHATTERPROOF 1* (*SHP1*) and *SHP2*) of the five down‐regulated *AG* targets in *ag‐11* were bound by AP2 *in vivo* and therefore potentially direct targets of *AP2* (Table S3). Thus, *AG* and *AP2* probably have direct and antagonistic effects on the transcription of these genes.

### *AP2* antagonizes *AG* activity in controlling floral determinacy

**Figure 6:**
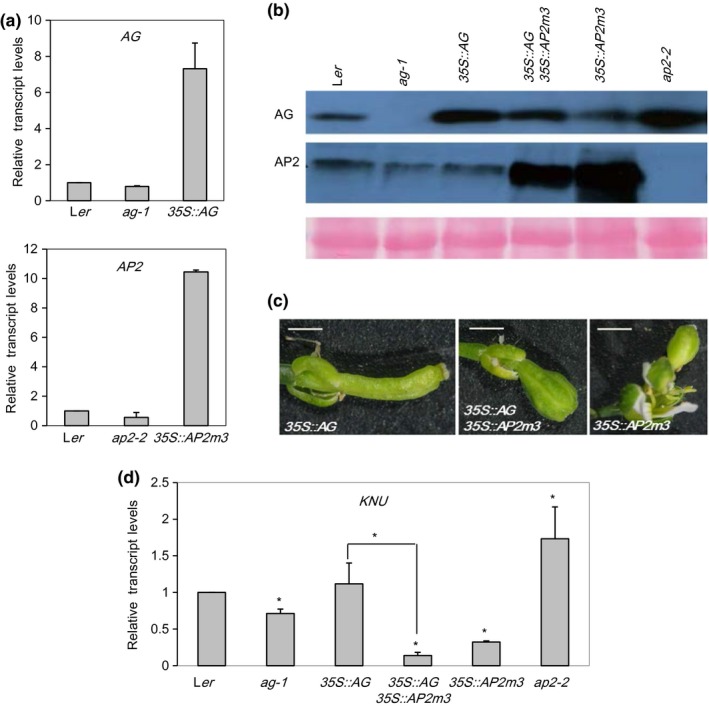
*APETALA2* (*AP2*) antagonizes *AGAMOUS* (*AG*) activity in controlling floral determinacy in *Arabidopsis thaliana*. (a) Real‐time reverse transcription−polymerase chain reaction (RT‐PCR) analysis of *AG* and *AP2* transcript levels in inflorescences of the indicated genotypes. (b) Western blot analysis of AG and AP2 protein levels in inflorescences of the indicated genotypes. Ponceau staining shown below was used as a loading control. (c) Floral phenotypes of the indicated genotypes. Bars, 2 mm. (d) Real‐time RT‐PCR analysis of *KNUCKLES* (*KNU*) transcript levels in inflorescences of the indicated genotypes. The asterisks directly above the genotypes indicate statistically significant changes relative to wild type (L*er*). The asterisk between *35S::AG* and *35S::AG 35S::AP2m3* indicates significant differences between these two genotypes. For (a) and (d), transcript levels were normalized to *UBIQUITIN* 5. Error bars indicate SD from three technical replicates.

To further establish that *AP2* antagonizes *AG* activity rather than inhibiting *AG* expression, we examined whether *AP2* affects floral determinacy when *AG* transcription is rendered independent of *AP2*. *AP2* is known to repress *AG* transcription in the outer two whorls through the large second intron of *AG* (Bomblies *et al*., 1999; Deyholos & Sieburth, 2000; Dinh *et al*., 2012). We expressed *AG* cDNA (thus devoid of the second intron) from the cauliflower mosaic virus 35S promoter. A transgenic line showing higher levels of *AG* RNA (Fig. 6a) and protein (Fig. 6b) was chosen for further analysis. This line exhibited the expected phenotypes, such as reduced plant stature and an absence of petals in the flowers (Fig. 6c) (Mizukami & Ma, 1992). To determine whether *AP2* could antagonize *AG* in the inner two whorls, it was necessary to express *AP2* there. But *AP2* is normally repressed in the inner two whorls by the microRNA miR172 (Chen, 2004). Thus, we expressed an miR172‐resistant version of *AP2* (*AP2m3*) under the 35S promoter (Chen, 2004). A transgenic line with increased *AP2* RNA (Fig. 6a) and protein (Fig. 6b) levels was chosen for further analysis. This line exhibited loss of floral determinacy (Fig. 6c), as previously reported for the overexpression of miR172‐resistant *AP2* (Chen, 2004; Zhao *et al*., 2007). To test whether *AP2* is functional in the presence of *35S::AG*, we crossed *35S::AP2m3* to *35S::AG*. In *35S::AG 35S::AP2m3* flowers, there were higher levels of both AP2 and AG proteins than in wild type. The gynoecia of *35S::AG 35S::AP2m3* flowers were bulged and present on top of elongated gynophores; the phenotype resembled that of the *35S::AP2m3* line but differed from that of the *35S::AG* line used in the cross (Fig. 6c). Therefore, *AP2* was able to antagonize the activity of *AG* even when *AG* was not under transcriptional repression by *AP2*.

*KNU* is the only gene known to act downstream of *AG* to promote floral determinacy (Sun *et al*., 2009). *AG* activates *KNU* expression at stage 6, and *KNU* in turn represses *WUS* expression (Sun *et al*., 2009). We investigated whether *AP2* also regulates *KNU* expression and, if so, whether it exerts the opposite effect to that of *AG*. We examined *KNU* RNA levels in the *ag‐11*,* ag‐11 ap2‐35* and *ag‐11 ap2‐43* RNA‐seq data. *KNU* transcript levels were increased in *ag‐11 ap2‐35* and *ag‐11 ap2‐43* relative to *ag‐11* (Fig. 5c), indicating that *AP2* represses *KNU* expression. We also compared *KNU* expression in wild type, *ap2‐2* and *35S::AP2m3*. Consistent with a repressive role of *AP2* on *KNU* expression, *KNU* expression was higher in *ap2‐2* and dramatically reduced in *35S::AP2m3* relative to wild type (Fig. 6d). To address whether *AP2* antagonizes *AG* in terms of *KNU* expression regulation, we compared *KNU* expression in *35S::AG* and *35S::AG 35S::AP2m3*. *KNU* expression was similarly low in *35S::AP2m3* and *35S::AG 35S::AP2m3*, suggesting that *AP2* overexpression overcame the presence of high levels of AG protein (Fig. 6d). These findings show that *KNU*, a key target of *AG* in floral determinacy, is also regulated by *AP2* but in the opposite way.

## Discussion

Efforts to understand the mechanisms of stem cell regulation in plants have largely focused on the SAM, which maintains its stem cell population throughout the life of a plant. Floral meristems undergo genetically programed termination of stem cell fate and this process is coordinated with other programs of flower development, such as floral organ formation and fruit development. Thus, floral meristems offer a great model with which to understand stem cell maintenance and termination as well as the interplay between stem cell activity and other developmental processes. From a practical point of view, adjusting the timing of floral stem cell termination could influence fruit size, as floral stem cell termination is coupled to carpel primordia formation.

**Figure 7:**
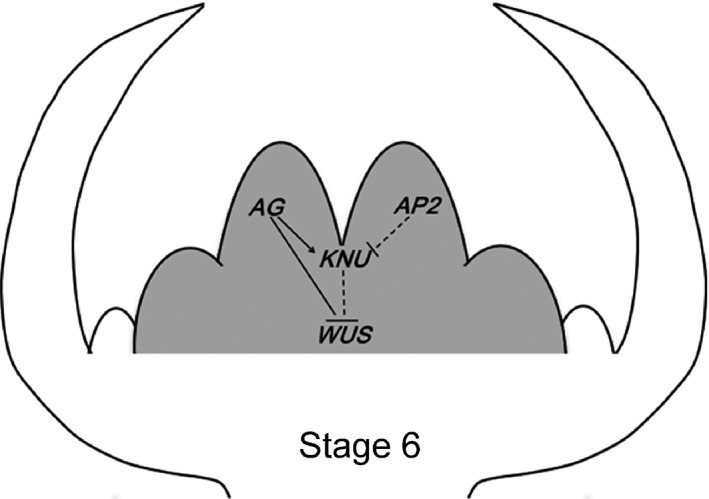
A model of *AGAMOUS* (*AG*) and *APETALA2* (*AP2*) in floral meristem determinacy in *Arabidopsis thaliana*. *KNUCKLES* (*KNU*) is the only gene known to act downstream of *AG* to promote floral determinacy, and it is used here as an example of a gene co‐regulated by *AG* and *AP2*. *KNU* expression is turned on at stage 6 in flower development, and it represses *WUSCHEL* (*WUS*) expression. *AP2* antagonizes *AG* by repressing the *AG* target gene *KNU*. Therefore, *AP2* could serve as a brake in the feed‐forward loop consisting of *AG*,*KNU* and *WUS*. Solid arrows, direct effects; dashed arrows, relationship may not be direct. Note that *AG* and *AP2* probably also act on other genes in the control of floral determinacy. As shown by RNA‐seq (Fig. 5), many genes involved in early flower development were found to be regulated by *AG* and *AP2* in opposite ways.

In *A. thaliana*, the termination of floral stem cell fate is accomplished by the repression of the stem cell maintenance gene *WUS* by *AG* at stage 6 of flower development when the carpel primordia have been formed. A feed‐forward loop consisting of *AG*, its target *KNU*, and *WUS*, a target of both *AG* and *KNU*, is probably at work to terminate floral stem cell fate (Fig. 7). In addition to *AG* and *KNU*, many other genes have been shown to participate in the repression of *WUS* (Bowman & Smyth, 1999; Carles *et al*., 2004; Zhao *et al*., 2007; Prunet *et al*., 2008; Ji *et al*., 2011; Liu *et al*., 2011; 2014a,b; Yumul *et al*., 2013). By contrast, how *WUS* expression is maintained in flower development is largely unknown. We showed that overexpression of *AP2* in flowers, achieved through the expression of miR172‐resistant *AP2*, leads to prolonged *WUS* expression and indeterminate floral meristems (Zhao *et al*., 2007). However, this evidence based on *AP2* overexpression was insufficient to establish a normal function of *AP2* in floral determinacy and maintenance of *WUS* expression.

In this study, we provide loss‐of‐function evidence that *AP2* maintains *WUS* expression in the floral meristem and promotes floral stem cell fate. Two *ap2* alleles with early stop codons were isolated as suppressors of the floral determinacy defects of *ag‐11*. The prolonged *WUS* expression in *ag‐11* was suppressed by the two *ap2* mutations, suggesting that *AP2* promotes *WUS* expression. *AP2* is well known for its role in floral organ identity specification in the outer two floral whorls, where it represses *AG* transcription and promotes the formation of sepals and petals. Here, we show that *AP2* also has a role in the center of the flower, where it promotes stem cell maintenance. Another study also revealed a role of *AP2* in fruit development (Ripoll *et al*., 2011). Therefore, *AP2*'s function in flower development is not restricted to the outer two whorls.

Another important conclusion from this work is that *AP2* antagonizes *AG* in floral determinacy not through the repression of *AG* transcription, as it does in the outer two whorls. We show that the two *ap2* loss‐of‐function alleles suppress *ag‐11* without affecting *AG* RNA levels in the center of the flower where floral stem cells reside. We also show that *AP2* overexpression can compromise floral determinacy even when *AG* is overexpressed from the 35S promoter. Therefore, *AP2* must be able to antagonize the function of *AG* either directly or indirectly. We show that *AP2* reduces the expression of *KNU* even in the *35S::AG* background. Therefore, *AP2* could exert its antagonistic effects on *AG* by repressing the *AG* target gene *KNU*. *AP2* could serve as a brake in the feed‐forward loop consisting of *AG*,* KNU* and *WUS* (Fig. 7). Note that *KNU* is a direct target of *AG* (Sun *et al*., 2009), but it is not known whether *AP2* acts directly on the *KNU* locus.

Finally, this study revealed a remarkable and rather unexpected antagonistic relationship between *AG* and *AP2* at the genomic scale. The altered expression of 25–35% of the 357 DE genes in *ag‐11* was affected in the opposite direction by the two *ap2* loss‐of‐function mutations. Among the 357 genes, 22 are bound by AG *in vivo* and are probably direct *AG* targets. Ten of the 22 *AG* direct target genes were found to be co‐regulated by *AG* and *AP2* in opposite directions. Therefore, *AP2* has a profound and previously unappreciated effect on *AG*'s transcriptional output.

## Author contributions

X.C., L.X. and Z.G. planned and designed the research. Z.H., T.S., B. Z., R.E.Y. and X.L. performed experiments. C.Y. analyzed genomics data. X.C. and Z.H. wrote the manuscript.

## Supporting information

Please note: Wiley Blackwell are not responsible for the content or functionality of any Supporting Information supplied by the authors. Any queries (other than missing material) should be directed to the *New Phytologist* Central Office.

**Fig. S1** Genotyping of the *ag‐1* and *ag‐11* mutations in the F_1_ progeny of the cross *ag‐11* × *ag‐1*/+.
**Fig. S2** Phenotypes of the indicated genotypes.
**Fig. S3** RNA‐seq analysis of wild type (L*er*), *ag‐11*,* ag‐11 ap2‐35*, and *ag‐11 ap2‐43*.
**Table S1** Sequences of oligonucleotides used in this study
**Table S2** Floral organ counts and quantification of floral determinacy defects
**Table S5** The *P*‐value of overlaps between DE genes

**Table S3** Genes with differential expression between *ag‐11* and L*er*
**Table S4** Genes with differential expression between *ag‐11 ap2‐35* and *ag‐11* and between *ag‐11 ap2‐43* and *ag‐11*
