# Supplementary material for: APETALA2 antagonizes the transcriptional activity of AGAMOUS in regulating floral stem cells in Arabidopsis thaliana
Source: New Phytol. 2016 Sep 8;215(3):1197–209. doi: 10.1111/nph.14151 (PMC5342953; doi:10.1111/nph.14151)
Supplement: Supplementary file 1 — Fig. S1 Genotyping of the ag‐1 and ag‐11 mutations in the F1 progeny of the cross ag‐11 × ag‐1/+. Fig. S2 Phenotypes of the indicated genotypes. Fig. S3 RNA‐seq analysis of wild type (Ler), ag‐11, ag‐11 ap2‐35, and ag‐11 ap2‐43. Table S1 Sequences of oligonucleotides used in this study Table S2 Floral organ counts and quantification of floral determinacy defects Table S5 The P‐value of overlaps between DE genes [file NPH-215-1197-s001.pdf]

New Phytologist Supporting Information Figs S1–S3 and Tables S1, S2 & S5

Article title: *APETALA2* antagonizes *AGAMOUS* in the regulation of stem cell maintenance in *Arabidopsis* flower development

Authors: Zhigang Huang,Ting Shi, Binglian Zheng, Rae Eden Yumul, Xigang Liu, Chenjiang You, Zhihong Gao, Langtao Xiao and Xuemei Chen

Article acceptance date: 18 July 2016

The following Supporting Information is available for this article:

Fig. S1 Genotyping of the *ag-1* and *ag-11* mutations in the F_1 progeny of the cross *ag-11* x *ag-1*/+.

Fig. S2 Phenotypes of the indicated genotypes.

Fig. S3 RNA-seq analysis of wild type (L*er*), *ag-11*, *ag-11 ap2-35*, and *ag-11 ap2-43*.

Table S1 Sequences of oligonucleotides used in this study

Table S2 Floral organ counts and quantification of floral determinacy defects

Table S3 Genes with differential expression between *ag-11* and L*er* (separate Excel file)

Table S4 Genes with differential expression between *ag-11 ap2-35* and *ag-11* and between *ag-11 ap2-43* and *ag-11* (separate Excel file)

Table S5 The *P*-value of overlaps between DE genes

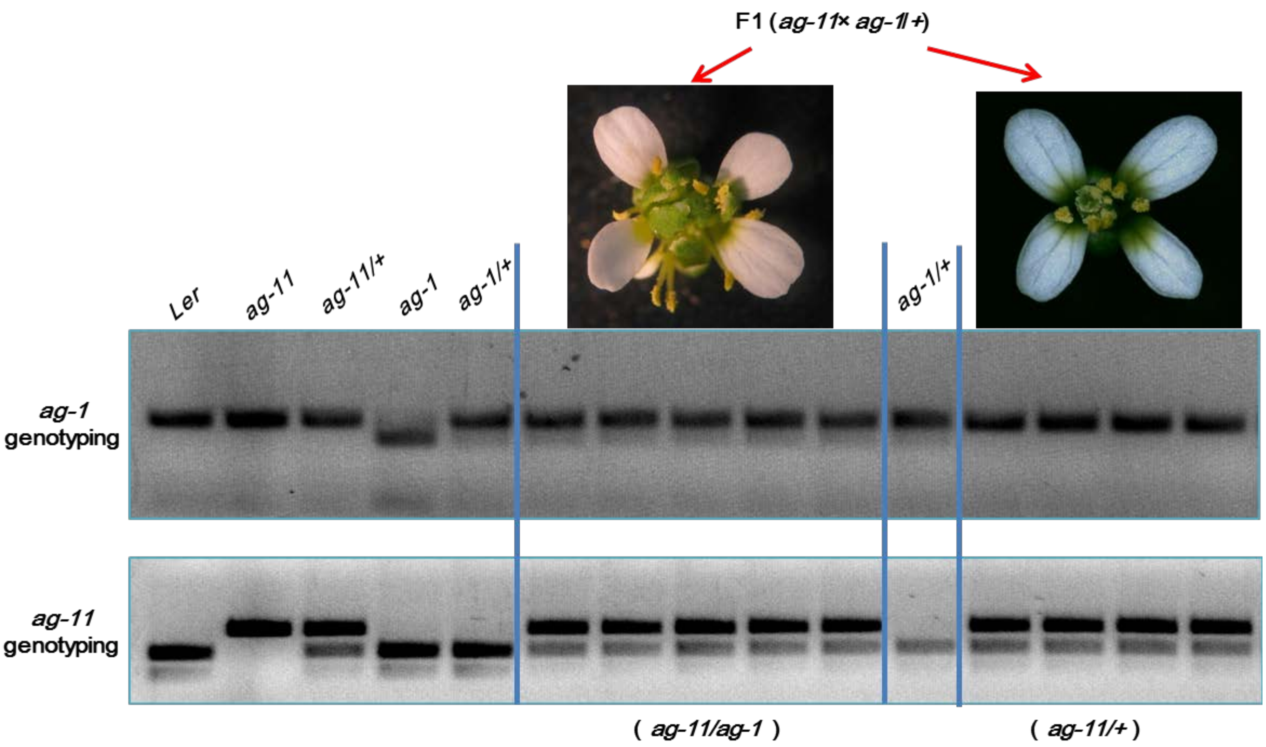

Fig. S1 Genotyping of the *ag-1* and *ag-11* mutations in the F1 progeny of the cross *ag-11* × *ag-1*/+. Plants of known genotypes (marked above the gel images) were used as controls for genotyping. '*ag-11*/+' and '*ag-1*/+' indicate plants heterozygous for *ag-11* and *ag-1*, respectively. The two floral images represent the phenotypes of the F_1 plants genotyped in the lanes below. These plants were first phenotyped and then genotyped, and their genotypes are indicated in parentheses below the gel images.

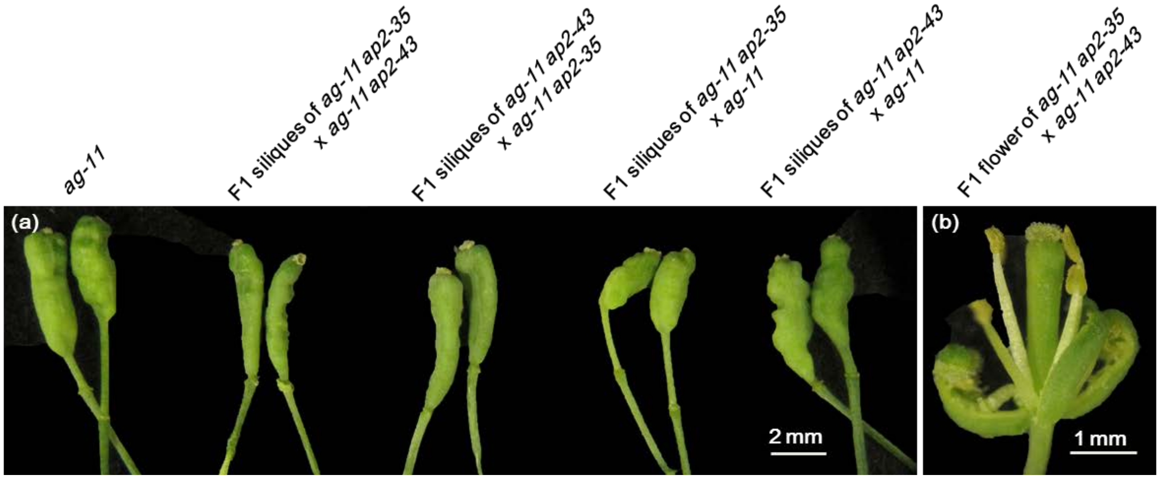

Fig. S2 Phenotypes of the indicated genotypes. Representative (a) siliques and (b) flower from plants of the indicated genotypes. F_1, F_1 progeny of the indicated crosses.

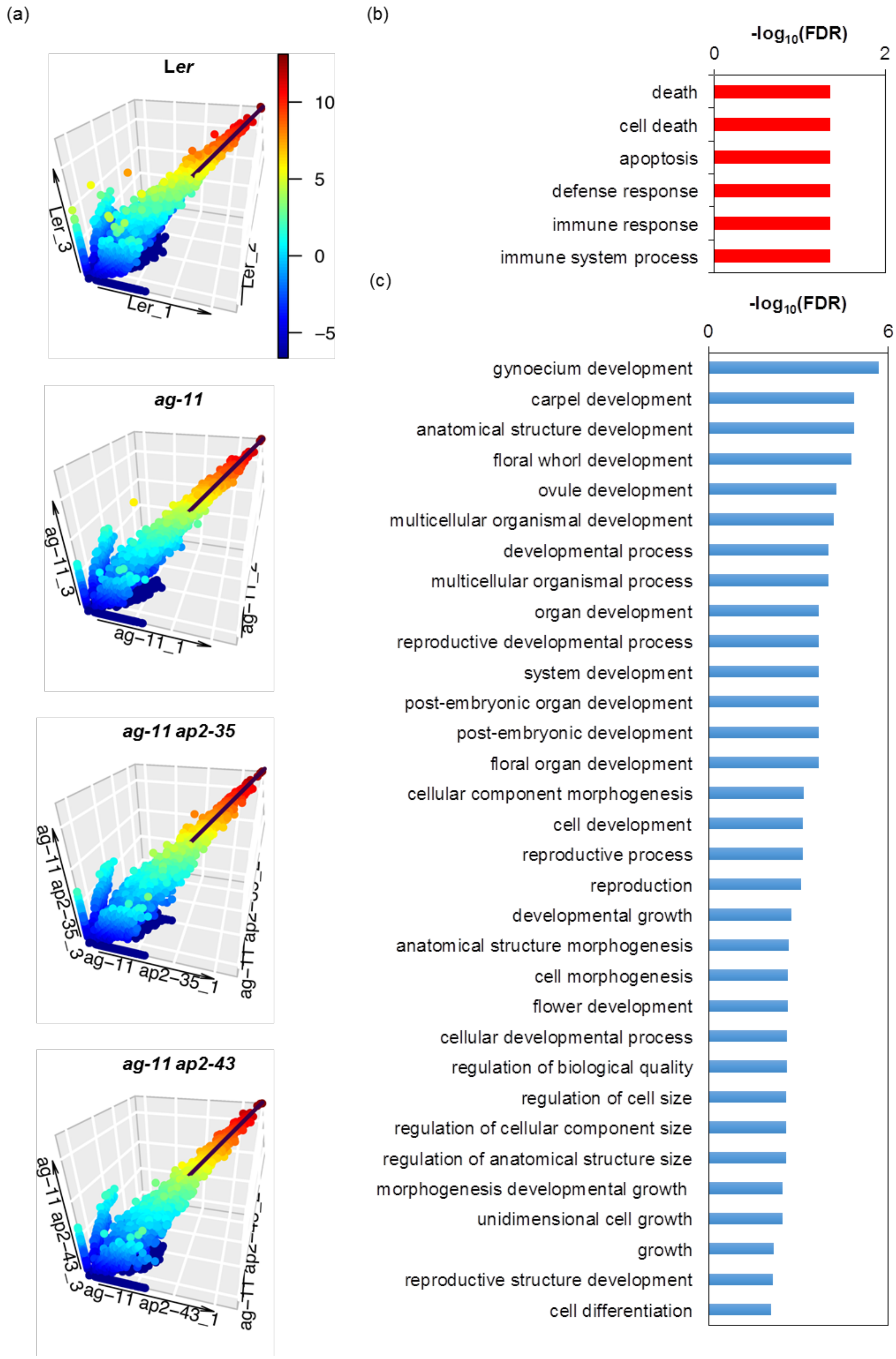
(a)
Ler
10
5
0
−5
Ler_1
Ler_3
ag-11
ag−11_1
ag−11_3
ag-11 ap2-35
ag−11 ap2−35_1
ag−11 ap2−35_3
ag-11 ap2-43
ag−11 ap2−43_1
ag−11 ap2−43_3
(b)
-log10(FDR)
0
2
death
cell death
apoptosis
defense response
immune response
immune system process
(c)
-log10(FDR)
0
6
gynoecium development
carpel development
anatomical structure development
floral whorl development
ovule development
multicellular organismal development
developmental process
multicellular organismal process
organ development
reproductive developmental process
system development
post-embryonic organ development
post-embryonic development
floral organ development
cellular component morphogenesis
cell development
reproductive process
reproduction
developmental growth
anatomical structure morphogenesis
cell morphogenesis
flower development
cellular developmental process
regulation of biological quality
regulation of cell size
regulation of cellular component size
regulation of anatomical structure size
morphogenesis developmental growth
unidimensional cell growth
growth
reproductive structure development
cell differentiation

Fig. S3 RNA-seq analysis of wild type (L*er*), *ag-11*, *ag-11 ap2-35*, and *ag-11 ap2-43*. (a) Scatterplots of gene expression from three biological replicates for each genotype to show reproducibility. Expression levels are represented by reads per kilobase per million mapped reads (RPKM). The three dimensions represent the three biological replicates. (b, c) Significantly enriched GO terms in (b) the upregulated and (c) downregulated genes in *ag-11* vs L*er*.

Table S1 Sequences of oligonucleotides used in this study

Name	Sequence	Note
JAGp75	CAATGTCTCCCAAAGAGCCCAGGAACTT	*ag-10* genotyping
JAGp76	GCAACAAGGCATATAGATTTAATTTG	
ag-11F	GCAATTGATGGGTGAGACGATAGGG	*ag-11* genotyping
ag-11R	CTTTTCTGCATGTAGTCGATTTCAGA	
ag-1F	GATATATTAACATATGTTGATAAATCACTTA	*ag-1* genotyping
ag-1F	ATAGAATTACCTTCTTGGATCGG	
UBQF	GGTGCTAAGAAGAGGAAGAA	Realtime RT-PCR
UBQR	CTCCTTCTTTCTGGTAAACGT	
AG-QF	CTCAGGAACTTGGAAGGCAGAT	Realtime RT-PCR
AG-QR	CTCTTTTCTGCATGTAGTCGATTTCA	
AP2-QF	GACGCACCACACCAAACACA	Realtime RT-PCR
AP2-QR	ATCTTGGTCCACGCCGACTC	
KNU-QF	CGTCCTCGCTAACTCTCCAC	Realtime RT-PCR
KNU-QR	ACGGATGAAACGGATCGTAG	
WUSSP6	GATTTAGGTGACACTATAGAATGACGACGGAGCAA ATCAAAA	*WUS* probe PCR
WUST7	TGTAATACGACTCACTATAGGGAGACGTAGCTCAA GAGAAGC	

Table S2 Floral organ counts and quantification of floral determinacy defects

**Genotype**	**Sepals**	**Petals**	**Stamen**	**Carpels**	**Internal organs (%)***	***n***
ag-11	4 ± 0	4 ± 0	5.7 ± 0.8	2.6 ± 0.6	100%	30
ag-11 ap2-35	na	na	5.3 ± 1.1	2.2 ± 0.5	16.7%	30
ag-11 ap2-43	na	na	5.2 ± 1.1	2.1 ± 0.4	13.3%	30

Values indicate the average and ± SD. na, not applicable. *Percentage of gynoecia with internal floral organs.

Table S5 The *P*-value of overlaps between DE genes

		L*er* vs *ag-11*	
		Downregulated genes	Upregulated genes
ag-11 vs. *ag-11 ap2-35*	Upregulated genes	2.20E-16	/
	Downregulated genes	/	3.13E-08
ag-11 vs. *ag-11 ap2-43*	Upregulated genes	1.49E-12	/
	Downregulated genes	/	9.78E-08
